# Bilateral strio-pallido-dentate calcinosis (Fahr’s disease): report of seven cases and revision of literature

**DOI:** 10.1186/s12883-016-0693-1

**Published:** 2016-09-08

**Authors:** Elisabetta Savino, Cecilia Soavi, Eleonora Capatti, Massimo Borrelli, Giovanni B. Vigna, Angelina Passaro, Giovanni Zuliani

**Affiliations:** 1 Department of Medical Sciences, Section of Internal and Cardiopulmonary Medicine, University of Ferrara, Via Savonarola n°9, 44100 Ferrara, Italy; 2 Azienda Ospedaliero-Universitaria S. Anna Ferrara, Ferrara, Italy

**Keywords:** Case report, Fahr’s disease, Basal ganglia calcification, Neurodegeneration

## Abstract

**Background:**

Fahr’s disease is rare a neurodegenerative idiopathic condition characterized by symmetric and bilateral calcifications of basal ganglia, usually associated with progressive neuropsychiatric dysfunctions and movement disorders. The term “Fahr’s syndrome” is used in presence of calcifications secondary to a specific cause, but the variability of etiology, pathogenesis, and clinical picture underlying this condition have raised the question of the real existence of a syndrome. Several classifications based on the etiology, the location of brain calcifications and the clinical presentation have been proposed. Here we describe seven clinical cases of basal ganglia calcifications, in order to search for pathognomonic features and correlations between clinical picture and imaging findings.

**Cases presentation:**

The patients came to our attention for different reasons (most of them for memory/behavior disturbances); all underwent neuro-psychologic evaluation and neuro-imaging. All patients showed variable degrees of deterioration in cognitive function; anxiety and depression were frequent too, and resistant to treatment in all cases. Less frequent, but severe if present, were psychotic symptoms, with different grade of structure and emotional involvement, and always resistant to treatment. We observed only few cases of extrapyramidal disorders related to the disease itself; anyway, mild extrapyramidal syndrome occurred quite frequently after treatment with antipsychotics.

**Conclusion:**

Based on these findings we discourage the use of the term “Fahr’s syndrome”, and suggest to refer to Idiopathic or Secondary basal ganglia calcification. Unlike early onset forms (idiopathic or inherited), the clinical presentation of late onset form and Secondary basal ganglia calcification seems to be really heterogeneous. Case–control studies are necessary to determine the actual significance of basal ganglia calcification in the adult population and in the elderly, in cognitive, physical and emotional terms.

## Background

*Fahr’s disease, Bilateral striopallidodentate calcinosis* [1], and *Idiopathic basal ganglia calcification* [2] are three of the more than 30 names used to identify a rare neurodegenerative condition characterized by the presence of bilateral calcification of the basal ganglia and other parts of the brain [3]. This condition has a wide range of possible clinical manifestations, usually marked by neuropsychiatric symptoms (i.e. dementia, schizophrenia-like psychosis, mood disorders), and extrapiramidal movement disorders [3]. Fahr was the first author who, despite a few previous case-reports by others [4, 5], described in 1930 the case of a demented patient with movement disturbances, whose autopsy revealed *vascular non atherosclerotic calcifications* in centrum semiovale and striatum [6].

For years, the terms *Fahr’s disease* and *Fahr’s syndrome* have been used indistinctly to identify different clinical conditions characterized by basal ganglia calcification. Nowadays, several authors use the term *syndrome* referring to clinical cases, symptomatic or not, in which a specific cause can be identified at the basis of brain calcification [7]. Nevertheless, there is no agreement about the use of the term “Fahr’s syndrome”, since, under this name, are grouped a very wide range of conditions, with different etiologies, pathogeneses, and different clinical phenotypes, raising the question of the real existence of such a syndrome. In fact, a study conducted in 2007 found that among 1569 healthy subjects, 0.8 % presented basal ganglia calcifications at CT scan, apparently without any symptoms, thus inducing the authors to consider them intracranial “physiological” calcifications [8]. More recently, irrespective of the etiology, Manyam proposed a classification based on the anatomical site in which calcium and other minerals depose, including 1. striopallidodentate calcinosis, 2. bilateral striopallidal (basal ganglia) calcinosis, and 3. bilateral cerebellar calcification [3]. In this paper we report seven consecutive cases of patients with basal ganglia calcification detected from a series of 750 patients referring to our Clinic for the study of Cognitive Diseases or to our Internal Medicine Ward from January 2003 to December 2013. Our purpose was, in a group of patients with non-genetic forms of basal ganglia calcinosis, to evaluate whether a clinical picture corresponding to neuroimaging could be detected and, consequently, whether the use of the term “syndrome” may be correct.

## Case presentation

### Case 1

M.G., a 73 year old man, came to our attention after the instruction of mandatory medical treatment, disposed to the police after reports of inadequate behavior. After repulsing his wife and daughter, five years before, he had been living alone, in a state of total neglect. His medical history was positive for type 2 diabetes, and a previous transient ischemic attack. No family history of neurologic nor psychiatric disturbances was reported. The physical examination revealed only poor self-care and bradykinesia. The findings at neuropsychological testing are summarized in Table 1.

**Table 1:** Principal characteristics of the seven subjects with Fahr’s disease

Case	Gender	Age	Diagnosis	Risk factors	Classification by Manyam (and other neuroimaging findings)	Psychotic symptoms	Cognitive impaiment	Other Psychiatric disorders	Extrapyramidal disorders	Other neurologic abnormalities	Response to neuroleptics
1	Male	73	Primary Late onset	(cerebrovascular disease ?)	SPD calcinosis (+ left, frontal malacic area, leukoaraiosis, cortical atrophy)	Severe: well-structured paranoid delusions of robbery and conspirancy, with strong emotional involvment	Severe, progressive: memory, language, orientation, calculation, attention (MMSE: 19/30; MODA: 88/100)	Apathia,	Bradykinesia Later, secondary hypertonia, diskinesias, tremor, gait disturbances		Poor
2	Male	37	Secondary Early onset	Head injury	SP calcinosis	Severe: poorly structured paranoid delusions of body transformation and greatness, auditory hallucinations, aggressiveness	Mild: language, comprehension, abstract reasoning, visual-motor ability. (WAIS: below lower limit)	Flattening of affectivity	Late, secondary (facial and limb diskinesias)	Amimia, fatuous expression	Poor
3	Male	54	Primary Late onset		SP calcinosis (+ mild cerebellar atrophy)	Absent	Mild: memory	Anxious-depressive syndrome focused on health problems	Absent	-	-
4	Female	48	Secondary Early onset	Head injuries, Hyperparathyroidism	SP calcinosis (+ mild cerebellar atrophy)	Absent	Mild: attention, memory, logical reasoning, language, visual-spatial skills.	Anxious-depressive syndrome	Absent	Inexhaustible glabellar reflex	-
5	Female	63	Primary Late onset		SPD + occipital calcinosis (+ frontal leukoaraiosis; left ponto-cerebellar cistern meningioma)	Absent	Very mild: sustained and divided attention	Mild focus on health condition	Absent	-	-
6	Female	73	Secondary Late onset	Hyperparathyroidism	SP calcinosis (worsened at follow-up with development of moderate atrophy, leukoaraiosis and left parietal cortical infarction)	Progressive: visual hallucinations, then aggressiveness, paranoid delusions	Progressive: memory, orientation. (initial MMSE: 24.7/30, then progressive worsening).	Major depression	Early, progressive (worsening tremor of superior limbs, then gait disturbances)	Palmo-mental reflex	Partial
7	Female	82	Secondary Late onset	Hyperparathyroidism, previous stroke?	SPD calcinosis (+ diffuse atrophy)	Progressive: visual and auditory hallucinations, then aggressiveness	Progressive, severe: memory, orientation, attention (MMSE 19.5/30)	Depression	Early (diffuse hypertonia, resting and intentional tremor, gait disturbances)	Inexhaustible glabellar reflex, bilateral Babinski reflex	Good

Legend: *MMSE* mini mental state examination, *MODA* Milan overall dementia assessment, *SP* strio-pallidal, *SPD* strio-pallido-dentate, *WAIS* Wechsler Adult Intelligence Scale

**Fig. 1:**
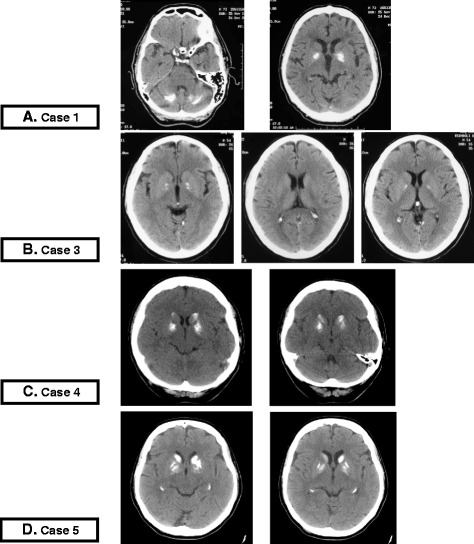
CT scan imaging of Cases 1,3,4 and 5. **a** Case 1. CT scans: extensive bilateral calcification of pallidus nuclei of basal ganglia and dentate nuclei of cerebellum; Hypodense malacic aspect in left frontal lobe. **b** Case 3. CT scans: mineral hyperdense deposits in bilateral pallidus nuclei without other area of altered density. Small enlargement, age-related, of frontal cerebral sulci due to atrophy. **c** Case 4. CT scans: hyperdense simmetric calcific deposits in pallidus nuclei and caudate nuclei of both sides. **d** Case 5. CT scans: hyperdense bilateral calcific deposits in the head of caudate nuclei, putamen and pallidus nuclei

Blood tests, comprehensive of calcium and phosphate, were normal. Computed Tomography (CT) scan (Fig. 1-a) and Magnetic Resonance Imaging (MRI) findings are summarized in Table 1. An antipsychotic treatment was started (quetiapine up to 200 mg/day) with poor response; after discharge behavior disorders recurred, inducing forced hospitalization in a nursing home. He displayed extrapyramidal symptoms, strong structured delusions, and worsening of dementia.

### Case 2

D.C., a 37 years old man, came to our attention for psychiatric evaluation to assess his capacity of discernment and his social dangerousness, after receiving a complaint for theft. The only anamnestic element was a head injury occurred 11 years before. After this trauma, his personality had changed with aggressiveness, recurrent migraine with auditory/olfactory hallucinations. A treatment with haloperidol had been tried, suspended because of scarce effect on psychotic disturbances and appearance of extrapyramidal symptoms. An extensive bilateral calcification of globus pallidus was revealed by CT scan. The findings of psychiatric examination are summarized in Table 1.

### Case 3

E.F., a 54 years old man, came to our observation for weight loss, weakness, and hyporexia. He had a psychiatric history of “somatoform autonomic dysfunction”, and had been followed by local Psychiatric Service during the previous years, and treated with selective serotonin reuptake inhibitors (SSRI) at maximum dose with inadequate results. His anamnesis was positive for coronary heart disease, hypertension, dyslipidemia, hyper-omocysteinemia, and monoclonal gammopathy of undetermined significance. Except for the rapid decrease in body weight, the physical and neurologic examination was unremarkable, as well as blood exams, abdominal ultrasound, and chest X-rays. Gastroscopy showed the presence of bulbar erosive duodenitis. The results of his psychiatric evaluation are summarized in Table 1. A CT scan revealed the presence of bilateral hyperdense calcium deposits in globus pallidus, and slight dilatation of the pericerebellar fluid space because of atrophy (Fig. 1-b-). A treatment with sertraline up to 150 mg/day, together with aripiprazole (15 mg/day) was started, with slight improvement in mood and clinical conditions.

### Case 4

B.S., a 48 years old woman, came to our attention sent by her psychiatrist for a neuro-psycological evaluation. She had a family history of vascular dementia, and her medical history was positive for hypothyroidism, obesity, dyslipidemia, and hypertensive cardiopathy. After a car accident with brain injury occurred 10 years before, she had suffered from anxious-depressive syndrome; some years later, a second car accident with brain trauma occurred. She was treated with venlafaxine and quetiapine, with poor response. Physical and neurologic examination were unremarkable, except for mild frontal liberation signs. Blood test showed hyperparathyroidism (Parathyroid hormone: 83 pg/ml; normal range: 12–65 pg/ml) associated with hypovitaminosis D (vitamin D: 10.8 ng/ml; normal range: 20–120 ng/ml); serum calcium was within normal values (2.55 mmol/l; normal range: 2.15–2.55 mmol/l). A thyroid ultrasound examination was unremarkable, parathyroids were not visible. Brain CT scan (Fig. 1-c) and neuropsychological evaluation results are shown in Table 1.

### Case 5

B.M., a 63 years old woman, was admitted to our Day Hospital after two episodes of syncope. She denied seizures, bite, and sphincter relaxation, but reported tinnitus and gait instability lasting two months. For this reason she had already undergone several examinations showing mild atherosclerotic carotid disease, a phenotype II-b dyslipidemia, and a deficit of Vitamin D without calcium, phosphorus or parathyroid hormone alterations. Her anamnesis was positive for hypertension, thyroid nodules, smoking habit. She had an high level of education (university degree) and a family history of depression and dementia. Physical and neurological examinations were normal except for orthostatic hypotension. The findings at cerebral MRI and CT scan, and results from neuropsychological tests are summarized in Table 1 and shown in Fig. 1-d.

### Case 6

M.M., a 73 years old woman was referred to our clinic for memory complaints, episodes of time/space disorientation, visual hallucinations, and sleep disorders. At the time of first visit she was still totally independent in basic, and partially in instrumental activities of daily living (score 18/19). Her anamnesis was positive for hypertension, COPD (Chronic Obstructive Pulmonary Disease), and major depression. Her physical examination showed mild frontal liberation and extrapyramidal signs (see Table 1). Blood tests revealed increased parathyroid hormone with normal calcium levels. Brain CT scan showed important bilateral calcification of globus pallidus. Citalopram (20 mg/day), risperidone (2 mg/day), and oxazepam were prescribed. On follow-up her memory and movement disturbances gradually worsened with development of psychotic symptoms (see Table 1), and a second CT scan revealed the worsening of known calcifications and development of degenerative/vascular encephalopathy (see Table 1). Quetiapine (100 mg/day) and promazine (200 mg/day) were prescribed in place of risperidone, with partial response. She progressively lost her independence until refusing of feeding on, and died at the age of 81.

### Case 7

C.L., a 82 years old woman was admitted to our ward for a lipothymia. Her anamnesis was positive for hypertension, carotid atherosclerosis, previous stroke, and frequent falls in the last 5 years. Her familiar history was positive for dementia. Neurological examination revealed both pyramidal and extrapyramidal signs (see Table 1). At neurologic evaluations cognitive impairment, depressive symptoms, and hallucinations emerged. Blood test revealed vitamin D deficiency, with increased parathyroid hormone and hypercalcemia, and a brain CT scan showed diffuse atrophy, and basal ganglia calcification. Sertraline, nimodipine, and vitamin D supplementation were started, with poor response. The progressive worsening of hypertonia, resistant to L-dopa treatment, caused several further falls, with many bone fractures. She progressively loss independency, was institutionalized, and developed aggressive behaviour responsive to promazine.

## Revision of literature

### Epidemiology

The frequency of incidental finding of basal ganglia calcification during CT scan raised from an incidence of 0.24–0.75 % during the eighties [9–12], until 12.5 % in two thousands [13, 14]. The reason of this increase depends on one side on the wide spread of CT-scan, and, on the other side, on larger new diagnostic criterions (from minimal monolateral calcification isolated to globus pallidus to massive bilateral calcification [3]. Fahr’s disease is considered a rare disorder, even if its real prevalence is still unknown. The epidemiology of this disease is so hard to be obtained for several reasons: firstly for the plethora of names used indistinctly to identify different conditions, secondly for the complexity of diagnosis with a wide spectrum of clinical manifestations (from asymptomatic people, to severe cognitive and movement disturbances), finally because the clinical manifestations are reported in literature mainly as case reports or small series. For example, applying uniform criteria to 61 cases of literature and 38 cases seen in a registry, Manyam found that less than 70 % of subjects were symptomatic [15]. There are no data indicating the mean age of onset of the disease, nor suggesting a gender prevalence, even if in the cited work by Manyam the male–female ratio was 2:1 [15].

### Etiology and histopathology

Considering the etiology, *bilateral striopallidodentate calcinosis* can be subdivided into four forms, with first to third belonging to *Fahr’s disease*, that means lacking of secondary causes [1]: 1. Autosomal dominant; 2. Familial; 3. Sporadic; 4. Secondary. The autosomal dominant form involves different people in the same parental line (parents or offsprings of the proband), and seems associated mainly to chromosome locus 14q48 mutation (IBCG1) [16–19]. Familial form is diagnosed when many members of the same family are affected, but this can be accounted for by chance, and there are not the characteristics to consider it a genetic form [3]. Fahr’s disease is sporadic when only one person of a family is affected; of course, the lack of symptoms in other family members is not enough to consider sporadic a case, but adequate neuroimaging studies should rule out brain calcifications. It must be underlined that a certain degree of calcification of basal ganglia can be considered “physiological” with aging, over 50 years [20].

Secondary forms, called *Fahr’s syndrome* by some authors, have been associated with several diseases, including: A) Abnormalities in calcium and phosphate metabolism, mainly hyper- [21, 22], hypo- [23, 24] and pseudo-hypoparathyroidism [25, 26]; B) Brain infections, such as Acquired Immune Deficiency Syndrome (AIDS) [27], Epstein–Barr virus [28], and other meningoencephalitis [29]; C) tumors (mainly angiomas and cerebral gliomas) [30, 31]; D) systemic lupus erythematosus [32, 33]; E) previous head injuries [34]. Calcium deposition involves basal ganglia, thalami, dentate nuclei of cerebellum, and occasionally cortical and sub-cortical frontal, parietal and occipital regions connecting associative cortex to basal ganglia and thalami [35]. The involvement of such a complex system explains the possible presence of movement, cognitive, and behavioral/emotional disorders in affected patients [35]. Histological and chemical studied demonstrated that several minerals are involved in the disease, like iron, magnesium, aluminum, and zinc [3], but the exact pathophysiological pathway is still unknown. We can argue, firstly, that the anatomy of the local arterial system could determine a low blood flow, leading to a reduction of glucose metabolism [36]. Secondly, there could be a slowly progressive metabolic and inflammatory modifications that lead to calcium deposition [37]. Differently from atherosclerotic lesions, the intima is spared [38] and the deposits mainly affect adventitia and basal membrane of the small vessels and the perivascular spaces [39]. Neuronal degeneration and gliosis in the surrounding areas firstly involve astrocytes [40], initially causing hypertrophy and hyperplasia, and finely leading to necrosis [41]. The affected areas are characterized by the deposition of muchopolysaccharides and proteins, followed by calcium and phosphorus in form of hydroxyapatite [42]. The extravascular deposition of an acid mucopolysaccharide-alkalic protein complex was suggested as a mechanism for the secondary accumulation of calcium, with vascular membrane abnormalities responsible for the leakage of plasma-derived fluid as suggested by MRI studies [38]. Furthermore, calcium phosphate deposition would be facilitated by the high concentration of iron and alkaline phosphatase in these areas [43]. Finally, basal ganglia are known to be the target for many other mineral and non-mineral deposits, such as bilirubin in the newborn, methyl-phenyl-tetrahydropyridine (MPTP) and carbon monoxide in poisoning [3], maybe indicating an intrinsic vulnerability of this system.

### Clinical features

The clinical presentation of the disease is really variable with a wide range of clinical presentations, from the completely asymptomatic subject to the patient with severe extrapyramidal and neuropsychiatric disorders. As already said, Manyam reported symptomatic subjects as being about 68 % in a series of 99 patients [15]; in symptomatic patients, the most commonly reported symptoms were movement disorders (55 %), like Parkinsonism, chorea, tremors, dystonia, athetosis, oro-facial diskinesia), followed by cognitive impairment (39 %), speech disorder (36 %), cerebellar impairment (36 %), and psychiatric symptoms (31 %). Relatively rare were pyramidal signs, gait disorders, sensory changes, and pain [15]. No differences in clinical presentation were found comparing secondary forms and Fahr’s disease. Similarly, no specific clinical patterns were found to be associated with the localization of calcium deposits, with similar clinical features in striopallidodentate, striopallidal, and dentate calcinosis. The only aspect significantly influencing the clinical features of Fahr’s disease seems to be the age of onset (probably because of a different and yet completely unknown etiology). Based on this, three different clinical patterns are distinguished [44]: 1) a *very rare infantile form*; 2) an *early onset type*, developing symptoms in the thirties, and mainly characterized by schizophrenia-like psychosis, with a lower role of movement disorders; 3) a *late onset type*, with symptoms onset in the fifties, whose typical features are dementia and movement disorders (Table 2). Movement disorders usually present early in the *late onset form* (even if sometimes late development of movement disorders have been reported), while they appear late in the *early onset form*, probably when progressive neurodegeneration exceeds a certain threshold. Dementia, with the classical characteristics of the primary sub-cortical types [45–47], is typical of the *late onset type*, but in younger patients a secondary evolution to dementia has been reported too. The pathogenesis of cognitive impairment is related to the destruction of connection between basal ganglia and cortex, also leading to fronto-temporal atrophy, formation of cortical neurofibrillar aggregates and loss of neurons, both in cortex and basal ganglia [45]. Psychotic symptoms are characteristic of the *early onset form*, and an association was hypothesized between the severity of clinical expression and the extension of calcifications [48]. This form was considered by DSM-IV (fourth version of the Diagnostic and Statistical Manual of Mental Disorders) one of the “psychotic disorders due to a general medical condition”, and as other organic psychosis is characterized mainly by hallucinations and delirium, but also mood and attention may be affected. Psychotic symptoms include auditory, visual and olfactory hallucinations, ideas of reference and persecutory delusions [45, 46, 49, 50]. Typically, psychiatric symptoms poorly respond to antipsychotic treatment, and an increase in susceptibility to adverse reactions to these therapies was documented [44]. Finally, in both clinical forms of the disease, other neuropsychiatric symptoms may occur, such as mood disorders (mainly depressive symptoms) and anxiety disorders.

**Table 2:** Principal clinical features of Fahr’s disease

	Fahr’s disease	
	Early onset	Late onset
Cognitive impairment/dementia	A cortical dementia may develop in advanced stages	Usually starts with a subcortical dementia
Psychotic disorders	Organic psychosis (basal ganglia calcification); delusion has syndromic pervasiveness behavioral, is moderately structured and organized, has low emotional participation, varied content. Abnormalities of perception are rare.	Organic psychosis (dementia): delusion is poorly structured and organized, override the contents of jealousy, poisoning, and persecution. Abnormalities of perception are frequent.
Mood disorders	May be associated to psychotic symptoms. Depressive disorders are prevalent towards maniacal ones.	Always associated with cognitive impairment, may precede symptoms as prodromes. Prevailing depression, irritability, hyper-emotionality, apathy
Anxiety disorders	Possible association between Fahr’s disease and obsessive-compulsive disorder.	Possible association between Fahr’s disease and obsessive-compulsive disorder. They can also be associated to cognitive impairment
Other neuro psychiatric/cognitive disorders	Possible attention’s disorders.	Progressive alteration of cognitive functions (attention, language, memory, constructive abilities, etc.)
Extrapyramidal movement disorders	In advanced stages of the disease.	May be present since the onset.
Response to therapy	Poor sensibility to neuroleptic treatment; high susceptibility to side effects.	Poor response to any type of symptomatic therapy. Frequent side effects.

### Prognosis

*Fahr’s disease* is characterized by a progressive and extremely variable course; for this reason, the prognosis is really variable and hard to predict. The neurodegenerative evolution of the disease was confirmed in two recent studies, in which brain CT scans demonstrated the development of cerebral atrophy in some affected patients [15, 51].

### Diagnosis

The diagnosis of Fahr’s disease is based on the presence of brain calcification on CT scan, together with the clinical manifestations, in absence of demonstrated alterations in calcium and phosphate serum levels and metabolism. However, because of the frequency of asymptomatic patients, the incidental diagnosis in patients undergoing brain CT scan for other reasons is possible. When parkinsonism is associated to dementia and cerebellar signs, obtaining a CT scan might be helpful [3]; when brain imaging is positive for bilateral striopallidodentate calcification, obtaining serum calcium and parathormone levels should help differentiating Fahr’s disease from hypoparathiroidism, which is the major differential diagnosis [3], leading to the diagnosis of secondary basal ganglia calcification. As regards secondary forms, the diagnosis is made, independently of the presence of symptoms, when cerebral calcifications are present at brain imaging, and a specific factor able to cause them is demonstrated. Among the neuroradiologic exams, brain CT is more sensitive than traditional MRI in the detection of calcium deposits [52], but the recently introduced SWI (Susceptibility-weighted imaging) MRI seems to be more sensitive for early changes [53, 54]; another useful tool is the 99mTehexamethyl-propylenamine oxime (99mTc-HMPAO) single proton emission computed tomography (SPECT)[55], revealing markedly decreased perfusion involving the basal ganglia bilaterally and cerebral cortices [56].

No marked alterations are found at neurophysiologic studies in Fahr’s disease patients: excluding the possible finding of minor abnormalities at brainstem auditory-evoked potentials, all other neurophysiologic exams give completely normal results [3]. Finally, decreased cerebrospinal fluid levels of calcium were detected in one study [57], but a confirmation is needed yet.

### Therapy

There is no therapy able to limit the progression of basal ganglia calcification in patients with Fahr’s disease. Moreover, despite a specific approach to all etiological identified factors, no improvement was demonstrated in secondary forms. In this context, at present time management and treatment strategies mainly focus on symptomatic relief, and are strictly related with the clinical features.

## Conclusions

In the present work we describe the clinical presentation of seven subjects with basal ganglia calcification. By comparing these clinical cases with current Literature we observed that actual studies don’t allow conclusions about real clinical features of basal ganglia calcification, in relation to qualitative and methodological limitations. The only exception is represented by inherited Fahr’s disease, in which the early calcium deposition in nucleus pallidus and striatum is inevitably related to severe, although heterogeneous, clinical manifestations. Moreover, it must be pointed out that the very few anatomical pathology studies show a late neuronal involvement; therefore, it is not possible to relate in any way the morphological findings obtained by CT scan with the real impairment of the anatomic pathways concerned. Several observations can be made considering the seven, heterogeneous, presented cases.

Patient of Case 1, was affected by *strio-pallido-dentate calcinosis.* This is probably a case of *Fahr’s disease* with severe primitive calcifications, even if a form secondary to cerebrovascular disease cannot be excluded. However, the clinical picture favors the hypothesis of *Late onset Fahr’s disease*, since well-structured delusions with emotional involvement are very rare in vascular dementia [58]. Extrapiramidal disorders developed two years after the first observation: they might represent late symptoms of the disease or side effect of antipsychotic treatment. The disappointing response to this treatment, and the possible development of side effects, are in favour of Fahr’s disease [44].

Case 2 was affected by *strio-pallidal calcinosis* [3], probably this was a case of Secondary BGC (Basal Ganglia Calcification) due to the previous brain injury. As reported for early onset forms, the predominant symptom was the psychotic disorder. Also mild cognitive impairment is a typical symptom of BGC, even if in early onset type it usually appears later. Unfortunately, no information about the neurologic examination before starting antipsychotic therapy was available; however at the time of evaluation the patient was amimic: in lack of other elements this could suggest a Parkinsonism. Finally, the unsuccessful response and hypersensitivity to antipsychotic therapy supports SBCG diagnosis [44].

Case 3 presented with a *strio-pallidal calcinosis* [3] too, but the later beginning of the symptoms configures a *late onset idiopathic* form, although we don’t actually know the duration of the psychiatric disturbance before the patient was taken in charge. The preponderant symptom was the anxious-depressive syndrome resistant to drugs. The observation of memory deficit suggests the presence of cognitive impairment, even if a complete neuropsychological evaluation was not available. However, the relatively lower impact of cognitive deficit compared with mood disorder is typical of the early onset forms. Besides basal ganglia calcification, the mild atrophy showed at CT scan could explain, at least in part, the cognitive deficits.

Case 4 presented a *strio-pallidal calcinosis* [3], in this case due to a probable secondary BGC. A mood disorder was the prevalent symptom, even if a mild cognitive impairment was also detected. Two probable etiologic elements must be considered: severe hypovitaminosis D with secondary hyperparathyroidism and brain injury. Alteration in calcium/D vitamin metabolism can be at the basis of BGC [21, 22], but symptoms develop only after the brain injury, which seems to act as a trigger in this case. Probably, the brain injuries accelerated the evolution of the pathology that otherwise would have manifested as a late onset form. Again, the depressive syndrome was resistant to antipsychotic/antidepressant therapy at maximum dose, suggesting that BGC may induce resistance to antidepressant treatment, in addition to cognitive decline (cases 3–4), and head trauma (case 4).

Case 5 presents a *strio-pallido-dentate* and occipital *calcinosis* [3], probably of idiopathic origin (Fahr’s disease). Despite a quite extensive calcification, symptoms were really mild, and the diagnosis was incidental. At neuropsychologic evaluation very mild cognitive deficits emerged: this could represent the outset of a *late onset* form, commonly characterized by dementia [46, 47], even if vascular dementia could not be excluded. Finally, BGC can be secondary to the small brain tumor [30, 31]; usually, gliomas and angiomas are involved, while no association with meningiomas have been reported in literature.

Case 6 presented a *strio-pallidal calcinosis* [3] probably secondary to hyperparathyroidism. On the opposite of case 5, this patient initially had a mild-moderate burden of calcification, but symptoms were quite severe from the beginning. This *late onset* form was characterized by progressively worsening cognitive deficits associated with psychotic disturbances partially responsive to therapy. Movement disorders were present from the beginning and continued after suspension and change of treatment. Cognitive impairment was attributable to brain calcifications since it progressed to severe dementia before the appearance of vascular lesions and atrophy.

Finally, case 7 presented a *strio-pallido-dentate calcinosis* [3] probably due to secondary hyperparathyroidism. The patient showed a late onset form with early cognitive and motor dysfunction and later appearance of behavior disturbances. The brain atrophy and the responsiveness of behavioral disturbances to treatment may suggest the disturbances were secondary to degenerative encephalopathy, rather than to BGC. Another possibility is to consider the brain atrophy itself as a consequence of BCG, as neurodegeneration has been reported to occur later in the course of the disease in some cases [15, 51]. The hypertonia unresponsive to L-dopa could be due to BGC or to cerebrovascular disease, suggested by the anamnestic presence of previous stroke.

The conclusions of our report are limited by the lack of anatomical pathology studies evaluating the real burden of calcification, and by the incomplete information we obtained about some of our cases. However, Fahr’s disease is a quite rare disorder, and by comparing 7 cases (one with each other and with literature) we could suggest some interesting conclusions:

1. Cognitive impairment

Even with a variable severity, it seems to be a constant finding in patients with basal ganglia calcification; in particular, the two domains most commonly involved in our experience seem to be memory and attention. This observation might result from the fact that basal ganglia play a role in maintaining the integrity of cognitive functions. Alternatively, the calcification of basal ganglia may be a marker of other alterations related to cognitive impairment, such as reduction of cerebral blood flow, atrophy, ischemic sub-cortical lesions. Anyway, our findings strongly argue against the lack of cognitive symptoms in these type of patients.

2. Anxiety and depression

They are common in patients with basal ganglia calcification, but less than the cognitive decline. In our experience, depression in BGC is resistant to pharmacologic therapy. Anxiety and depression might be secondary to the impairment of the serotonin and dopamine pathways involved in the limbic system. Depression, frequently associated to cognitive impairment, might also represent a phenomenon preceding the onset of dementia, as often happens in the natural history of this disease [59].

3. Psychotic disorders

They may be associated to basal ganglia calcification. When present, they are usually severe, with different grade of structure and emotional involvement; nevertheless, we didn’t observe a clear pattern of presentation of psychotic symptoms (different organic background?). Moreover, in agreement with literature, psychosis poorly responds to specific treatment.

4. Extrapyramidal disorders

We observed only few cases (4/7) of extra pyramidal disorders probably related to the disease; in particular, significant gait disturbances and tremor were developed by the three older individuals. Anyway, a mild extra pyramidal syndrome might have developed also as a side effect of antipsychotic drugs.

In conclusion, while Fahr's disease with early onset seems to present fairly homogeneously, the clinical presentation of late-onset forms of Idiopathic/Secondary BGC seems to be much more heterogeneous, as evidenced by the presented clinical cases and the data from literature.

Based on these results, we discourage the use of the term “Fahr’s syndrome” to describe secondary causes, and prefer referring to them as “Secondary Basal Ganglia Calicification”.

Our data don’t allow to address the border phenotype between physiological calcinosis and putative pathological calcinosis of basal ganglia, since in our case series all of seven cases present some pathological aspects. This result might come from a “selection bias”: working in a Clinic for the study of Cognitive Impairment, most of the patients we evaluate come to our attention because of cognitive/behavioural/mood disturbances. However, we found basal ganglia calcifications in about 0.9 % of our patients, and this result is not different from the 0.8 % found in the general population by Daghighi et al. [8]. In our case series, basal ganglia calcification never seems to be an isolated and clinically insignificant finding, but always underlies cognitive/psychiatric/motor disturbances. Of consequence, although in some cases basal ganglia calcification might be an occasional finding, some cases (unknown percentage) present with overt clinical symptoms, and the degree of calcification at neuroimaging does not necessary correlate with the severity of clinical picture. Of consequence, we suggest that all subjects with the finding of BGC undergo a screening of cognitive performance and mood state. It could be correct to consider affected by “Fahr’s disease” only people in which BGC have a pivotal role in determining the symptoms, while all conditions in which calcifications are the marginal consequence of different insults to basal ganglia should be labelled as BGC.

## Abbreviations

99mTc-HMPAO: 99mTehexamethyl-propylenamine oxime
AIDS: Acquaired Immune Deficiency Syndrome
BGC: Basal ganglia calcification
COPD: Chronic Obstructive Pulmonary Disease
CT: Computed Tomography
DSM-IV: Fourth version of the Diagnostic and Statistical Manual of Mental Disorders
MMSE: Mini Mental State Examination
MODA: Milan Overall Dementia Assessment
MPTP: Methyl-phenyl-tetrahydropyridine
MRI: Magnetic Resonance Imaging
SPECT: Single Proton Emission Computed Tomography
SSRI: Selective Serotonin Reuptake Inhibitors
WAIS-R: Wechsler Adult Intelligence Scale
